# The Recover Study: A Cross-Sectional Examination of the Relationship Between Ontario Parents’ Resilience and COVID-19-Related Stressors

**DOI:** 10.1177/10664807221123550

**Published:** 2022-09-04

**Authors:** Julia Yates, Jennifer D. Irwin

**Affiliations:** 1 Faculty of Health Sciences, The University of Western Ontario, London, Ontario, Canada; 2 School of Health Studies, Faculty of Health Sciences, The University of Western Ontario, London, Ontario, Canada

**Keywords:** resilience, stressors, COVID-19, parents, school-aged children

## Abstract

Resilience, or the ability to bounce back despite facing adversities, may influence parents’ abilities to handle the multitude of parent-specific COVID-19-related challenges that have faced them. This cross-sectional study examined (1) the relationship between parents’ resilience and their COVID-19-related family stressors; (2) parents’ perceptions of their greatest stressors throughout the pandemic; and (3) non-school-related challenges and their resultant impact on parents’ and children's resilience. Via an online survey, data was collected from 63 parents (M_age_ = 37.09; 82.54% female). A significant relationship was found between parents’ resilience and both their COVID-19-related stressors and family stressors. Parents described stressors challenging their resilience, including impacts on their mental health, managing occupational and educational responsibilities, social isolation, and economic setbacks, while also noting the impacts of social isolation, missing extracurricular activities, and lacking routines for their children. Overall, Ontario parents high in resilience are likely better positioned to adapt to pandemic-related stressors.

## Introduction

The World Health Organization (WHO) declared COVID-19 a pandemic on March 11th, 2020 (WHO, 2020). By March 14th, the Ontario Ministry of Education had closed all schools. Across the province, this initial closure lasted 14 weeks (i.e., until June 30th; Canadian Institute for Health Information, 2021). As cases and deaths resulting from the COVID-19 virus continued to fluctuate, public health measures to mitigate the spread of the virus also varied, resulting in an oscillation between in-person and online learning for students across Ontario (Gallagher-Mackay et al., 2021). This fluctuation resulted in Ontario elementary school students being out of school longer than those in any other Canadian province or territory (Subramanian, 2021). Although province-wide elementary school closures have happened only three times thus far, many regions and individual schools have continued to experience closures due to high COVID-19 cases (Gallagher-Mackay et al., 2021). These ongoing public health measures have been essential to slow the spread of the virus, and have also reportedly resulted in social isolation, psychological and economic distress, and an increase in unhealthy coping habits among some adults (Coller & Webber, 2020; Gadermann et al., 2021; Gassman-Pines et al., 2020). Specifically, parents with children under the age of 18 and living at home have been at a disproportionate risk of experiencing worsened mental health due to the pandemic (Gadermann et al., 2021). Worrying about their children's education and heightened stress related to navigating child-minding and home-based/online schooling while continuing to work themselves have been especially challenging for parents (Gadermann et al., 2021). Although a quick internet search will bring up hundreds of links to advice for parents trying to cope with stress during the pandemic, there is currently a paucity of research exploring what has been especially helpful to parents, although Cusinato et al. (2020) proposed that a parent's resilience may be a key variable influencing their ability to effectively deal with pandemic-related challenges.

Resilience can be defined as a dynamic process in which psychosocial and environmental factors interact to enable an individual to survive, grow, and thrive despite exposure to adversity (Munoz et al., 2017; Prime et al., 2020). In a recent cross-sectional study conducted on parents (*n* = 107; 80.4% female) in Singapore, Lim et al. (2021) found that higher resilience was related to lower depression, anxiety, and stress among this population following the initial COVID-19 lockdown in March of 2020. Similarly, while investigating child adjustment in Spain during the pandemic, Romero et al. (2020) noted that the resilience of parents (*n* = 1049; 89.6% female) exerted a direct and negative effect on their general perceived distress resulting from the pandemic, such that higher resilience among parents indicated lower levels of perceived distress. This relationship between resilience and perceived distress also positively impacted depression and anxiety screenings, resulting in higher resilience among parents being associated with a lower incidence of depression and anxiety (Romero et al., 2020). Given the outlined relationships between resilience and psychological distress, it is possible that parents’ resilience may be impacted by COVID-19-related stressors.

For many parents, a myriad of stressors may be challenging their resilience during the COVID-19 pandemic. Parents may continue to worry about the impacts that education disruptions have had on their children, with learning gaps caused by closures far surpassing what was originally predicted heading into the lockdown phases (Subramanian, 2021). Despite the return to in-person learning for many, the prolonged impacts of the pandemic on students, and the resultant stress facing parents remains concerning. The impact of these stressors in addition to the variation in school closures experienced by parents and families may have contributed to further distress among parents in the already trying times of the ongoing pandemic. While studies have tried to quantify the experiences of parents (e.g., Cusinato et al., 2020; Dominguez-Alvarez et al., 2020; Laufer & Bitton, 2021), there is currently a dearth of evidence surrounding parents’ perceptions of these stressors and how they may be impacting their resilience or the resilience of their children.

While the impact of the COVID-19 pandemic on children's schooling has been an obvious and substantial challenge for many parents, it is not the only one. Compared to age-similar, non-parent adults, parents have experienced stronger reductions in their social support and heightened financial stress (Cluver et al., 2020; Gadermann et al., 2021; Lee & Ward, 2020). In a recent study conducted on parents (*n* = 1451; 72.3% female) in China, Ren and colleagues (2020) found that less social support was significantly related to increased parenting stress during the pandemic. A qualitative study conducted by Dawes et al. (2021) echoed these findings, as parents in this study (*n* = 29) described the increased stress and exhaustion resulting from navigating pandemic-related changes without their usual support networks. The financial impacts of the pandemic on families have also been emphasized by many (e.g., Brewer & Gardiner, 2020; Rodrigues et al., 2021). More Canadian families with children (aged < 18) living at home reported financial strain in the first month of the pandemic than families without children, at 54% versus 41%, respectively (Kaddatz, 2020). At the same time, and perhaps influenced by this financial strain, parents also reported mood, mental health, and sleep disturbances (Kaddatz, 2020). Parents have undoubtedly faced many struggles throughout the pandemic and, whether stemming from school closures or non-school-related factors, their first-hand accounts of these challenges are severely lacking within the literature.

Although the COVID-19 pandemic has likely exacerbated challenges to parents, the specific ways in which Ontario parents have been impacted remain unknown. While the resilience of parents during the pandemic has been investigated by several, to the authors’ knowledge no studies have investigated the resilience of Ontario parents during the COVID-19 pandemic. Given the variations in pandemic experiences around the world and within Canada (e.g., a longer period of school closures in Ontario compared to other provinces and territories), it is plausible that Ontario parents may have experienced different challenges to their resilience than parents in other provinces of Canada. Moreover, while some studies have explored parents’ resilience and psychological distress during the pandemic, to date, no studies have examined parents’ resilience in relation to COVID-19-specific stressors. While still valuable, these studies were not focused specifically on whether the stress experienced by parents was due to COVID-19, or a result of other contextual factors; therefore, the relationship between resilience and specific COVID-19-related stressors remains largely unexplored. Gaining insights into the experiences of parents will allow for a better understanding of the breadth and severity of pandemic-induced stressors as determined by the population members themselves. This timely study is designed to address this research gap to help understand Ontario parents’ capacity to survive and thrive throughout the pandemic, while also providing insights into the experiences of Ontario parents and children. Implications of this research could serve to benefit families of over one million elementary school-aged children who have been impacted by the COVID-19 pandemic in Ontario (Ontario Ministry of Education, 2021).

### Purpose

The three-fold purpose of this study was to examine: (1) the relationship between Ontario parents’ resilience and their COVID-19 family stressors; (2) Ontario parents’ perceptions of the greatest stressors challenging their resilience throughout the pandemic; and (3) non-school-related challenges that have surfaced throughout the pandemic and their resultant impact on the resilience of Ontario parents and children (as perceived and reported by parents).

## Methods

This cross-sectional study was conducted from January 17th–February 14th, 2022. The study received ethics approval from the host institution's Non-Medical Research Ethics Board. The data that support the findings of this study are available from the corresponding author, Julia Yates, upon reasonable request.

### Study Procedures

Study participants were English-speaking Ontarians residing in the province and a parent of at least one child in an English-speaking kindergarten (Junior or Senior), Primary (grades 1–3), or Junior (grades 4–6) elementary school (i.e., public, private, and religious). Participants were recruited via online advertisements (i.e., Kijiji, Facebook, Instagram, Twitter, and LinkedIn) through a total of 153 ads posted by the research team. Upon clicking the online advertisement and QR code, interested parents were directed to the study's online survey hosted on qualtrics^XM^ (Qualtrics, Provo, UT). The study's letter of information and eligibility criteria were outlined first, and upon providing informed and voluntary consent, the survey instructions and content appeared. To diminish social desirability bias, honesty demands (per Bates, 1992) were employed. That is, the instructions included the statement: “There are no right or wrong answers, we are only looking for the answers that are true for you.” After completing the survey, participants had the option to enter a draw for one of three $60 grocery gift cards.

### Measures

The online survey consisted of a short demographic questionnaire, the previously validated 10-item Connor-Davidson Resilience Scale (CD-RISC-10; Connor & Davidson, 2003) and the COVID-19 Family Stressors Scale (CoFaSS; Prime et al., 2021), as well as three open-ended questions to more deeply explore parents’ perceptions and experiences of their pandemic-related stressors.

#### Demographic questionnaire

The demographic questionnaire consisted of 12 items pertaining to participants’ gender, age, ethnicity, education, employment, income, children, and living situations.

#### Connor-Davidson resilience scale

The CD-RISC-10 was previously validated for use among parents (Cronbach's α range from 0.75 to 0.91; Campbell-Sills et al., 2009; Ye et al., 2017) and consists of 10 statements that describe aspects of resilience. Items correspond to flexibility, sense of self-efficacy, ability to regulate emotion, optimism, and cognitive focus/maintaining attention under stress (Connor & Davidson, 2020). Participants were asked to indicate how much they agreed with the statements as they applied to them over the last month on a five-point scale ranging from 0 (i.e., not at all true) to 4 (i.e., true nearly all of the time). Examples of statements include: “I am able to adapt when changes occur” and “I am not easily discouraged by failure.”

#### COVID-19 family stressors scale

The CoFaSS was recently validated for family-based research (Cronbach's α range from 0.68 to 0.82; Prime et al., 2021) and consists of 16 items that measure COVID-19-related psychological stressors. The CoFaSS includes three subscales: (1) family stress, which includes seven items (i.e., items 9–15) related to family altercations and child management; (2) income stress, where five items (i.e., items 1–3 and 6–7) relate to income, debts, and job loss; and (3) chaos stress, with four items (i.e., items 4–5, 8, and 16) relating to access to supplies, crowded shopping areas, and news coverage (Prime et al., 2021). Participants were asked if, since the onset of the COVID-19 pandemic in March 2020, any of the changes described in the tool had occurred in their households by responding to the statements as either not true, somewhat true, or very true. Examples of statements include: “Could not access essential supplies (e.g., sanitizer, soap, toilet paper, etc.)” and “Inability to access educational materials for children.”

#### Open-ended questions

Three open-ended questions were co-created by the researchers to address the study's 2nd and 3rd purpose statements: (1) “Reflecting on your experiences during the COVID-19 pandemic, what, in your role as a parent, has been the single greatest stressor that has challenged your resilience?”; (2) “Reflecting on your experiences during the COVID-19 pandemic, what would you classify as the top three most influential, non-school-related challenges that have surfaced in your child(ren)'s life that has impacted their resilience?”; and (3) “In what ways have these non-school-related challenges that your child(ren) has/have experienced impacted your own resilience?”. To conceptualize these questions, a definition of resilience was provided on the questionnaire prior to the questions: “In this study, resilience is conceptualized as a dynamic process in which psychosocial and environmental factors interact to enable an individual to survive, grow, and thrive despite exposure to adversity.”

### Data Analysis

All analyses were completed in RStudio (version 1.3.1090; RStudio Team, 2020). Measures of central tendency and dispersion were computed for demographic characteristics and each tool. Participants were included in analyses if they completed seven or more of the items on the CD-RISC-10 and 12 or more of the items on the CoFaSS (i.e., < 30% data missingness). If less than 30% of a participant's data was missing, a mean score of their completed answers was imputed for the remaining blank responses and they were included in analyses (per Dong & Peng, 2013).

#### Connor-Davidson resilience scale

Total scores on the CD-RISC-10 were calculated by summing the 10 items to yield a score ranging from 0 to 40, with higher scores indicating greater resilience, and lower scores indicating less resilience, or more difficulty in bouncing back from adversity (per Connor & Davidson, 2020). Following the work of Campbell-Sills and Stein (2007), the distribution of scores on this scale were organized into quartiles such that the lowest quartile scored between 0–29, the second between 30–32, the third between 33–36, and the top between 37–40.

#### COVID-19 family stressors scale

Total scores on the CoFaSS were calculated by summing the 16 items to yield a total score ranging from 16 to 48. Higher scores indicate parents facing increased COVID-19-related psychological stressors. Total scores on the family, income, and chaos stress subscales range from 7 to 21, 5 to 15, and 4 to 12, respectively, with higher scores indicating more stress in the corresponding aspect of family life (per Prime et al., 2021).

#### Relationship between resilience and COVID-19 family stressors

Pearson product-moment correlations were conducted to investigate the relationship between resilience and COVID-19 family stressors. A separate correlation was conducted between resilience and: (1) overall COVID-19 family stressors; (2) family stress; (3) income stress; and (4) chaos stress.

#### Open-ended response questions

To identify any emergent patterns, themes, and categories within the responses to the open-ended, inductive content analysis was conducted independently by two researchers (per Patton, 2005). To help ensure data trustworthiness and uphold qualitative rigor, several strategies were incorporated into data analysis, including analysis being completed separately by multiple coders, collectively solving any discrepancies and agreeing on main themes, and recording the process in detail to allow others the opportunity to re-create the process and determine if findings are transferable to other populations (per Guba & Lincoln, 1989).

## Results

### Demographics

The online survey was completed by 63 parents with a mean age of 37.09 years (*SD* = 5.64). Most parents identified as female (*n* = 52; 82.54%), of European descent (*n* = 32; 50.79%), and most frequently reported completing a college or university degree (*n* = 27; 42.86%) or higher (*n* = 19; 30.16%). Just over half of the parents were employed full-time prior to COVID-19 (*n* = 35; 55.56%) and at the time of completing the survey (*n* = 34; 53.97%), with working conditions for most remaining the same during COVID-19 (*n* = 20; 31.75%). The average annual household income was between $80,000 and $110,000 (*n* = 15; 23.81%). Most commonly, parents reported having two children (*n* = 28; 44.44%) and living in a two-parent household (*n* = 44; 69.84%). The mean age of the participants’ children was 7.01 years (*SD* = 3.47). See Table 1 for a detailed overview of the participants’ demographics.

**Table 1.** Demographic Information.

Participant Characteristics (*n* = 63)	*n*	%
Age, *M* (*SD*)	37.09 (5.64)	
Age of Children, *M* (*SD*)	7.01 (3.47)	
**Gender**		
Female	52	82.54
Male	4	6.35
Non-binary	1	1.59
**Ethnicity**		
African	2	3.17
Asian	2	3.17
Caribbean	1	1.59
European	32	50.79
Indigenous	4	6.35
Latin, Central, or South American	2	3.17
Mixed/multiracial	2	3.17
Other North American	8	12.70
Prefer not to answer	10	15.87
**Highest education achieved**		
Advanced degree	19	30.16
College or university degree	27	42.86
High school	6	9.52
Less than high school	1	1.59
Some college or university	3	4.76
**Employment status prior to COVID-19**		
Casual	3	4.76
Employed full-time	35	55.56
Employed part-time	9	14.29
Other	4	6.35
Unemployed	3	4.76
**Current employment status**		
Employed full-time	34	53.97
Employed part-time	7	11.11
Other	9	14.29
Unemployed	6	9.52
**Change in working conditions due to COVID-19**		
Other	5	7.94
Shifted from in-person to at-home	18	28.57
Shifted to a mix of in-person and at-home	13	20.63
Working conditions remained the same	20	31.75
**Average annual household income**		
< $30,000	1	1.59
$30,000–$59,999	9	14.29
$60,000–$79,999	11	17.46
$80,000–$110,999	15	23.81
$111,000–$150,000	3	4.76
> $150,000	11	17.46
**Number of children living in household**		
1	12	19.05
2	28	44.44
3	10	15.87
4	4	6.35
**Number of parents in household**		
1	11	17.46
2	44	69.84
Other	2	3.17

### Resilience

Based on responses from the CD-RISC-10, the mean resilience score of this sample was 27.44 (*SD* = 6.19). The scores of individual items can be found in Table 2.

**Table 2.** Descriptive Statistics of CD-RISC-10.

Item	Mean	Standard Deviation
1. I am able to adapt when changes occur.	2.92	0.90
2. I can deal with whatever comes my way.	2.97	0.91
3. I try to see the humorous side of things when I am faced with problems.	2.60	0.77
4. Having to cope with stress can make me stronger.	2.44	1.01
5. I tend to bounce back after illness, injury, or other hardships.	2.86	0.90
6. I believe I can achieve my goals, even if there are obstacles.	2.86	0.82
7. Under pressure, I stay focused and think clearly.	2.68	0.95
8. I am not easily discouraged by failure.	2.57	0.91
9. I think of myself as a strong person when dealing with life's challenges and difficulties.	2.99	0.81
10. I am able to handle unpleasant or painful feelings like sadness, fear, and anger.	2.73	0.96
Total	27.44	6.19

### COVID-19 Stressors

The mean total score on the CoFaSS scale was 29.11 (*SD* = 6.67). The mean scores on each domain of the CoFaSS across parents were 13.00 (*SD* = 3.44), 7.97 (*SD* = 2.94), and 8.14 (*SD* = 1.92) for the family, income, and chaos stress subscales, respectively. The average scores of each item can be found in Table 3.

**Table 3.** Descriptive Statistics of CoFaSS.

Item	Mean	Standard Deviation
**Family stress subscale**		
Experienced increased altercations with family members.	1.93	0.74
Experienced increased emotional withdrawal from family members.	2.00	0.76
Children have become harder to manage.	1.92	0.75
Inability to access educational materials for children.	1.65	0.74
More relationship conflicts with my partner (if I am in a relationship).	1.70	0.75
Difficulty developing a new family and/or personal routine.	1.94	0.74
Felt crowded in my living space.	1.86	0.82
Subscale total	13.00	3.44
**Income stress subscale**		
Significant decrease (over 10%) in household income.	1.19	0.84
Gone into financial debt.	1.38	0.633
Job disruption or loss (myself or my partner).	1.71	0.85
Applied for employment insurance or government assistance.	1.59	0.85
Became concerned about providing for family.	1.70	0.82
Subscale total	7.97	2.94
**Chaos stress subscale**		
Could not access essential supplies (e.g., sanitizer, soap, toilet paper, etc.).	1.54	0.67
Overwhelmed by the amount of COVID-19 news coverage.	2.41	0.66
Became stressed by crowded grocery stores and shopping centers.	2.21	0.72
Significant anxiety/panic about danger to myself or loved ones.	1.98	0.79
Subscale total	8.14	1.92
Scale total	29.11	6.67

### The Relationship Between Resilience and COVID-19 Family Stressors

Results from the Pearson product-moment correlations indicated evidence of a significant negative relationship between parents’ resilience and their overall COVID-19-related psychological stressors (*r* (61) = −0.27, *p* = .04; Table 4). More specifically, resilience was found to be significantly and negatively correlated with the family stress subscale (*r* (61) = −0.33, *p* = .009; Table 4). No significant relationships were found between resilience and income or chaos stress (Table 4).

**Table 4.** Correlations Between Resilience and COVID-19 Related Family Stressors.

Scale	*R*	*R^2^*	*df*	*p*
CD-RISC-10				
CoFaSS	−0.27	0.07	61	.04*
Family stress subscale	−0.33	0.11	61	.009**
Income stress subscale	−0.11	0.01	61	.40
Chaos stress subscale	−0.17	0.03	61	.19

*p < .05.

**p < .01.

### Open-Ended Response Questions

#### Greatest stressor challenging Ontario parents’ resilience

When asked what the single greatest stressor challenging their resilience was, parents were consistent in noting the following broad themes: (1) mental health impacts; (2) managing occupational and educational responsibilities; (3) lack of social interactions; and (4) economic impacts. Specifically, parents stressed the challenges of the constant uncertainty surrounding the pandemic and the resulting exhaustion. Further, the feeling of being responsible for their children's well-being was mentioned by many. Regarding occupational and educational responsibilities, parents noted the struggles of educational transitions and trying to find a balance between their seemingly ever-changing responsibilities throughout the pandemic. The lack of social interactions also challenged parents’ resilience in terms of having to navigate family relationships and attempting to maintain connections for their children. Finally, economic impacts were noted by many parents who stressed the challenges of dealing with job losses and barriers to accessing financial support. Table 5 presents illustrative quotes for each of these themes.

**Table 5.** Greatest Stressor Challenging Ontario Parents’ Resilience.

**Mental health impacts**
*Uncertainty* “The Unknown … Whether my son will be able to go to school, whether we will get covid again and we don't know who is vaccinated or not.” “Having to respond to uncertain circumstances in all facets of life—as a parent, spouse, employee, manager, daughter of aging parents to support wellness and health and safety.”
*Responsibility for children's well-being* *“*Guilt of not being a good enough parent.” “Seeing the mental and emotional health of my child deteriorate.”
*Exhaustion* *“*I’m spent. I’ve been solo parenting during the day, half of that time working full time from home and need a good, solid break from my children. It's been too much.”
**Managing occupational and educational responsibilities**
*Educational transitions* *“*The ever-changing lockdown, working from home, home school and all activities being done in the home.” “School … worrying about what kind of education my child is getting bouncing from in class to online learning.”
*Finding Balance* *“*Trying to balance a new full-time teaching job during a pandemic with two elementary-aged children and a toddler with food allergies and empty grocery store shelves.” “The hardest thing for me was the constant back and forth of online and in-person learning … My job is not easily transferable to work from home”
**Lack of social interactions**
*Navigating family relationships* *“*Being divorced and managing COVID protocols in a split-family situation” “Having to limit social interaction for our children. Less connection with family”
*Maintaining connections* *“*Balancing keeping kids safe but not isolated.”
**Economic impacts**
*Job losses* *“*My husband lost his job early in the pandemic and we were living in a very expensive city …. There's still significant childcare stress and worries about the kids being sent home for illness”
*Barriers to financial support* *“*Lack of access to funding for supports” “Funding available but long processing times.”

#### Non-school-related challenges impacting Ontario children's resilience

Parents reported the most influential, non-school-related challenges impacting their children's resilience as a lack of connection within both peer and family relationships, the absence of extracurricular activities, and a dearth of routine. Quotes illustrating these themes can be found in Table 6.

**Table 6.** Non-School-Related Challenges Impacting Ontario Children's Resilience.

**Lack of connection**
*Peer relationships* *“*Lack of friendships outside of the home, unaware of social norms outside of the home” “Difficulty making friends in a new city due to Covid closures and shut downs was very hard. They are very social kids”
*Family Relationships* *“*Missing family gatherings… and being with the immediate family 24/7” “Lack of family support due to isolating from loved ones” “A major shift in their relationships outside our immediate family: at various points we stopped and/or adjusted play time with other children, and they’ve had long stretches of time when we didn't see our extended family.”
**Absence of extracurricular activities** **“**The loss of extracurricular activities/disappointment when things shifted from in-person to virtual … a feeling of ‘missing out’” “Not being able to participate in their sport lessons—not know when this ends”
**Lack of routines** **“**My kids have become less motivated in many areas such as getting dressed, being social, sticking to a routine” “Lack of structure from adapting to the constantly changing situation, consuming COVID news, trying to work and parent consecutively. I think the kids sometimes felt like we had less time for them, even though we’ve spent more actual time together during these two years. More pressures on our attention.”

#### Impacts of children's non-school-related challenges on Ontario parents’ resilience

When asked how the above-noted challenges have impacted parents’ own resilience, some participants noted an increase in inner strength, specifically in relation to family connection/cohesion and personal growth. Parents also mentioned feelings of burnout resulting from feeling discouraged and an overall lack of time to spend on self-care activities, as well as prolonged impacts on their well-being. Finally, parents shared the impact of having to bear the weight of their children's emotions, rending many to experience a state of constant worry for their children while striving to make up for missed opportunities. Descriptive quotations of these findings can be found in Table 7.

**Table 7.** Impacts of Children's Non-School Related Challenges on Ontario Parents’ Resilience.

**Increased inner strength**
*Family connection and cohesion* *“*We have more conversation, more family time and we do a lot of activities together”
*Personal growth* *“*I’ve actually learned to be a lot more forgiving and open to change because my way to cope with stressors is not the same for my children and I must be supportive of their needs as well” “Challenged me to be present and patient when it was the last thing I felt I could do”
**Burnout**
*Discouraged* *“*I don't know how much longer I can do this. We got covid this week and I’m thankful my husband is home now too. I don't care anymore—eat whatever, watch a device, don't change into clothes. Just survive. No thriving” “Concern, anxiety, increased worry about the health, growth and well-being of my children has lowered my ability to handle it all”
*Lack of Self-Care Time* *“*I am burnt out. No time to myself” “We are on our own all the time with no one to help besides my spouse, I am worn out and need a break with no kids”
**Prolonged impacts on well-being** **“**It's been very hard to deal with everything that has been going on and it has impacted my mental health greatly. I constantly worried before but now it's a lot more” “I’ve had to prioritize things. The long-term stress has definitely impacted my resilience reserves”
**Bearing the weight of children's emotions**
*Constant worry* *“*My kids’ happiness directly impacts my happiness. When they are lonely I feel so heartbroken for them.” “It's made me feel sad, upset, and angry that they are missing out on years of their childhood that are precious and feeling hurt seeing them sad and worrying that they could become depressed”
*Striving to compensate for missed opportunities* *“*I feel like we have lost time and opportunities and now feel pressure on the longer-term impacts and need to compensate. Also feeling personally drained so hard sometime to give more at the end of a long day” “Increased pressure to entertain/manage kids at home—my bucket is always empty; - the world is a sad place, I fear for my kids and their future”

## Discussion

The purpose of this study was three-fold: (1) to examine the relationship between parents’ resilience and their COVID-19 family stressors; (2) to examine parents’ perceptions of the greatest stressors challenging their resilience throughout the pandemic; and (3) to assess non-school-related challenges that have surfaced throughout the pandemic and their resultant impact on the resilience of parents and children (as perceived and reported by parents).

### The Relationship Between Parents’ Resilience and COVID-19 Stressors

Although the average level of resilience for the study sample was low, it is interesting to note that it still seemed sufficiently robust to be related to lower COVID-19-related stress levels. However, it is not clear whether one variable predicts the other. That said, based on previous research (e.g., Cusinato et al., 2020; Laufer & Bitton, 2021; Romero et al., 2020) it is likely that parents’ resilience helped to mitigate the impacts of COVID-19-related psychological stressors. This finding is in line with previous work conducted on parents (*n* = 353; M_age_ = 42.15; 64% female) in Italy by Marzilli et al. (2021) who investigated resilience, stress, and mental health of parents of school-aged children (M_age_ = 9.28). The authors reported that parents’ distress due to COVID-19 was significantly and negatively associated with their resilience (*r* = −0.27, *p* < .01; Marzilli et al., 2021). Similar findings were noted in Lim et al. (2021) Singapore-based study exploring factors affecting parents’ psychological distress throughout the pandemic (*n* = 107; 80.4% female). The parents with high resilience scores were less likely to have scores indicating problematic levels of stress (OR = 0.97, *p* = .037) when compared to parents scoring lower on resilience (Lim et al., 2021). In the absence of support to help reduce the actual stressors experienced by parents during the COVID-19 pandemic, taken together, these findings suggest that bolstering resilience among parents is, perhaps, one way to counteract the effects of the stressors themselves.

The higher parents’ resilience levels, the less family stress they reported. There is a dearth of evidence regarding parental resilience in relation to family stress in the context of COVID-19; however, as outlined above, previous studies have purported a strong likelihood that parents’ resilience may serve as a protective factor against stress (e.g., Cusinato et al., 2020; Laufer & Bitton, 2021; Romero et al., 2020). While interpretations cannot be stated definitively, in the context of the current study, parents’ resilience may have contributed to lower levels of family-related stress. Although not considering the resilience of parents, Hwang et al. (2022) did study how Canadian families have been functioning (*n* = 254) in the context of COVID-19. Similar to the current study, these authors highlighted that family functioning is impacted by the COVID-19 pandemic. More specifically, problematic family functioning (e.g., difficulties problem solving, communicating) was noted in almost 80% of families in their sample (Hwang et al., 2022). While it is unknown whether parental resilience played a role in this problematic family functioning, given the significant negative correlation found in the current study between resilience and family stress, it is possible that Canadian parents could be experiencing low resilience during the COVID-19 pandemic. The relationship outlined above regarding resilience and family stress is similar to work published in Aivalioti and Pezirkianidis (2020) study of parents (*n* = 83; 76% female) living in Greece. Although conducted pre-pandemic, authors of this study on parental resilience and family well-being noted that those parents who reported high levels of resilience were more likely to report higher levels of family well-being compared to parents reporting low levels of resilience (Aivalioti & Pezirkianidis, 2020). Given the likelihood of high parental resilience contributing to lower family-related stress, optimized family functioning, and overall family well-being, it is plausible that supporting parents in bolstering their resilience through periods of adversity may support a ripple effect of positive outcomes among families. Ultimately, supporting parents such that they are not needing to draw upon their resilience reserves is a recommended top priority. When the available supports are insufficient to ameliorate the experience of adversity, incorporating evidence-based resilience-promoting strategies might be a suitable option.

Interestingly, there did not appear to be a relationship between parents’ resilience and COVID-19-related income stress. While it was hypothesized that the COVID-19 pandemic may have substantial impacts on the socioeconomic situation of families, this did not appear to be the case in our sample. One reason for these findings could be due to the high income reported by this sample (i.e., the majority reported an average annual household income between $80,000 and $110,999) compared to the median after-tax income of Ontarians in 2019, which was $66,600 (Statistics Canada, 2021). Moreover, most parents in this study reported living in a two-parent household, which may have further contributed to the high income reported by this sample given that lone-parent families in Ontario reported an almost $10,000 lower median after-tax income in 2019 compared to two-parent families (Statistics Canada, 2021). Based on these demographic characteristics, it may be possible that the current sample did not face a substantial change in income-related stress as a result of the COVID-19 pandemic; therefore, in this sense, resilience related to socioeconomic hardships may not have been required for this sample. In contrast to the findings of the current study, several studies have highlighted that the majority of parents in their samples (i.e., > 50%) have expressed financial worries and associated stressors that come along with their pandemic-related worries (Laufer & Bitton, 2021; Lim et al., 2021; Sorkkila & Aunola, 2021). Further, in a recent review, Prime et al. (2020) explored risks to families during COVID-19. These authors highlighted the pronounced economic reach of COVID-19 and the likelihood of many parents facing unprecedented increases in daily stressors (Prime et al., 2020). Possible reasoning for these discrepancies in the relationship between parents’ resilience and income-related stress may be the timelines during which each of these studies were conducted. While the aforementioned studies were all conducted during the initial months of lockdown caused by the COVID-19 pandemic, the current study was conducted almost two years into the pandemic. As such, it is possible that income-related stressors may have been experienced by this sample during the initial stages of the pandemic and may have leveled off since.

### The Greatest Stressors Challenging Parents’ Resilience

Parents in the current study described their pandemic-related stressors as both substantial and difficult to manage. Similar concerns about the challenges in coping with prolonged family and support disruptions were reflected in Guruge et al.’s (2021) qualitative study focused on the pandemic's prolonged upset for Toronto-based parents. Specifically, through semi-structured interviews, participants shared the impacts of COVID-19 on parenting, noting some of the greatest challenges being the prolonged duration at home causing a shift in family cohesion, the closure of schools placing increased pressure on parents to fill multiple roles (e.g., parents, spouses, employees, instructors), fear regarding the impacts of the pandemic on their children, and changing lifestyles and daily habits (Guruge et al., 2021). In a similar qualitative study conducted by Weaver and Swank (2020) on parents (*n* = 11; 82% female) in the United States, participants echoed the feelings of those in the current study regarding the difficulties of managing multiple roles and shouldering prolonged concern for their children's well-being. Specifically, participants expressed the difficulties of transitioning from in-person to online school, changing routines and priorities, the constant vacillating of emotions, and a loss of connection and support due to physical and social isolation (Weaver & Swank, 2020). Given the similar experiences highlighted by parent-focused studies, it is clear that parents have been experiencing resilience-requiring and -draining pandemic-induced stressors, including changing family dynamics, shifts in occupational and educational demands, and concerns surrounding the impacts of the pandemic on their children.

### The Impacts of Non-School-related Challenges on Parents and Children

The absence of extracurricular activities combined with the paucity of having connections with others that were especially concerning to parents in the current study, have been reported since the pandemic began. For example, in June 2020, Statistics Canada’s (2020) study of 32,000 parents revealed strong concerns about reduced social connections. Stemming from the cancelation of extracurriculars and social events, 71% of parents were concerned regarding socialization opportunities for their children, while 54% were concerned about their children's social isolation (Statistics Canada, 2020). Similarly, Szpunar et al. (2021) studied parents (*n* = 12; 91.7% female) and children (*n* = 9; M_age_ = 7.33) living in Ontario, with a specific focus on their physical activity and sport-related behaviors during the pandemic. Via semi-structured interviews, children noted challenges with being unable to see friends at extracurriculars as well as the closure of outdoor spaces and activity facilities (Szpunar et al., 2021). With the uncertainty surrounding the current pandemic mixed with the possibility of future ones, it seems imperative that public health plans to keep citizens safe also include creative programing for safe extracurricular activities to help support children's and parents’ resilience (e.g., ensuring all programing is supported such that every child and parent engaging in extracurriculars can be provided with high-quality masks immediately and at no cost, whenever required).

### Strengths and Limitations

There are notable strengths to this study. First, to the authors’ knowledge, this is the first study to investigate the relationship between Ontario parents’ resilience and their COVID-19 family stressors during the ongoing pandemic. In addition, the tools used to capture all quantitative data were previously validated among the targeted study population. Finally, the lived experiences of parents were also captured in this study, thus providing important contextual information to supplement the quantitative findings. While self-report measures lend themselves to social desirability (Callegaro, 2008), honesty demands, which help to encourage truthful responses, were employed to limit this risk (per Bates, 1992). Despite these strengths, there are also several limitations worth noting. First, the data collected was cross-sectional, thus leading to inherent weaknesses including an inability to make causal inferences between constructs or to investigate the temporal relations between variables (Wang & Cheng, 2020). And, although cross-sectional studies are valuable in determining the prevalence and studying the associations of multiple outcomes, they are also susceptible to recall bias whereby participants may not accurately recall previous experiences (Wang & Cheng, 2020). Finally, the lack of demographic diversity present in this Ontario sample limits the generalizability of the results. Most parents identified as females of European descent and high socioeconomic status. Given these characteristics of the sample, participants may have been experiencing fewer COVID-19-related stressors compared to others, such as those who are members of marginalized groups and/or navigating greater financial strain. Future studies may benefit from employing stratified sampling, resulting in a sample whereby various genders, ethnicities, and socioeconomic statuses are more strongly represented.

## Conclusion

Ontario parents high in resilience may be better equipped to adapt to pandemic-related stressors, including overall psychological stressors and family-related stress. Further, based on first-hand accounts of pandemic experiences, Ontario parents and children appear to be impacted by a myriad of challenges, many of which may be hindering their resilience. These impacts of the pandemic on Ontario parents and children must be taken into account when making future public health-related decisions. It is evident that parents and children could benefit from immediate and ongoing support throughout future periods of adversity.

## References

[bibr1-10664807221123550] Aivalioti I. Pezirkianidis C. (2020). The role of family resilience on parental well-being and resilience levels. Psychology (Savannah, Ga), 11(11), 1705–1728. 10.4236/psych.2020.1111108

[bibr2-10664807221123550] Bates B. L. (1992). The effect of demands for honesty on the efficacy of the Carleton skills-training program. The International Journal of Clinical and Experimental Hypnosis, 40(2), 88–102. doi:10.1080/00207149208409650 1582728

[bibr3-10664807221123550] Brewer M. Gardiner L. (2020). The initial impact of COVID-19 and policy responses on household incomes. *Oxford Review of Economic Policy, 36*(2020), S187–S199. 10.1093/oxrep/graa024 PMC7337863 40504196

[bibr4-10664807221123550] Campbell-Sills L. Forde D. R. Stein M. B. (2009). Demographic and childhood environmental predictors of resilience in a community sample. Journal of Psychiatric Research, 43(12), 1007–1012. 10.1016/j.jpsychires.2009.01.013 19264325

[bibr5-10664807221123550] Campbell-Sills L. Stein M. B. (2007). Psychometric analysis and refinement of the Connor-Davidson resilience scale (CD-RISC): Validation of a 10-item measure of resilience. Journal of Traumatic Stress, 20(6), 1019–1028. 10.1002/jts.20271 18157881

[bibr135-10664807221123550] Callegaro, M. (2008). Social desirability. In P. J. Lavrakas (Ed.), Encyclopedia of survey research methods (pp. 826-826). Sage Publications, Inc., 10.4135/9781412963947.n537

[bibr6-10664807221123550] Cluver L. Lachman J. M. Sherr L. Wessels I. Krug E. Rakotomalala S. Blight S. Hillis S. Bachman G. Green O. Butchart A. Tomlinson M. Ward C. L. Doubt J. McDonald K. (2020). Parenting in a time of COVID-19. The Lancet, 395(10231), e64. 10.1016/S0140-6736(20)30736-4 PMC7146667 32220657

[bibr7-10664807221123550] Coller R. J. Webber S. (2020). COVID-19 and the well-being of children and families. *Pediatrics, 146*(4), 1-3. 10.1542/peds.2020-022079 32873717

[bibr8-10664807221123550] Connor K. M. Davidson J. R. T. (2003). Development of a new resilience scare: The Connor-Davidson resilience scale (CD-RISC). *Depression and Anxiety, 18*(2), 71–82. 10.1002/da.10113 12964174

[bibr9-10664807221123550] Connor K. M. Davidson J. R. T. (2020). Scoring and interpretation of the Connor-Davidson Resilience Scale (CD-RISC).

[bibr10-10664807221123550] Cusinato M. Iannattone S. Spoto A. Poli M. Moretti C. Gatta M. Miscioscia M. (2020). Stress, resilience, and well-being in Italian children and their parents during the COVID-19 pandemic. International Journal of Environmental Research and Public Health, 17(22), 8297. 10.3390/ijerph17228297 33182661 PMC7696524

[bibr11-10664807221123550] Dawes J. May T. McKinlay A. Fancourt D. Burton A. (2021). Impact of the COVID-19 pandemic on the mental health and wellbeing of parents with young children: A qualitative interview study. *BMC Psychology, 9*(2021), 1-13. 10.1186/s40359-021-00701-8 PMC8672159 34911570

[bibr12-10664807221123550] Dong Y. Peng C. Y. J. (2013). Principled missing data methods for researchers. *SpringerPlus, 2*(222), 1-17. 10.1186/2193-1801-2-222 PMC3701793 23853744

[bibr131-10664807221123550] Dominguez-Alvarez, B., Lopez-Romero, L., Isdahl-Troye, A., Gomez-Fraguela, J. A., & Romero, E. (2020). Children Coping, Contextual Risk and Their Interplay During the COVID-19 Pandemic: A Spanish Case. *Frontiers in Psychology, 11*(101550902), 577763. 10.3389/fpsyg.2020.577763 PMC7772313 33391095

[bibr13-10664807221123550] Gadermann A. C. Thomson K. C. Richardson C. G. Gagne M. McAuliffe C. Hirani S. Jenkins E. (2021). Examining the impacts of the COVID-19 pandemic on family mental health in Canada: Findings from a national cross-sectional study. BMJ Open, 11(1), e042871. 10.1136/bmjopen-2020-042871 PMC7804831 33436472

[bibr14-10664807221123550] Gallagher-Mackay K. Srivastava P. Underwood K. , et al. (2021). COVID-19 and education disruption in Ontario: Emerging evidence on impacts. *Science Briefs of the Ontario COVID-19 Science Advisory Table, 2*(34), 1-36. 10.47326/ocsat.2021.02.34.1.0

[bibr15-10664807221123550] Gassman-Pines A. Ananat E. O. Fitz-Henley J. (2020). COVID-19 and parent-child psychological well-being. *Pediatrics, 146*(4), 1-9. 10.1542/peds.2020-007294 PMC7546085 32764151

[bibr16-10664807221123550] Guba E. G. Lincoln Y. S. (1989). Fourth generation evaluation. Sage Publications.

[bibr17-10664807221123550] Guruge S. Lamaj P. Lee C. Ronquillo C. E. Sidani S. Leung E. Ssawe A. Altenberg J. Amanzai H. Morrison L. (2021). COVID-19 restrictions: Experiences of immigrant parents in Toronto. AIMS Public Health, 8(1), 172–185. 10.3934/publichealth.2021013 33575415 PMC7870386

[bibr18-10664807221123550] Hwang P. Ipekian L. Jaiswal N. Scott G. Amirali E. L. Hechtman L. (2022). Family functioning and mental wellbeing impairment during initial quarantining for the COVID-19 pandemic: A study of Canadian families. *Current Psychology, 41*(1), 1-13 . 10.1007/s12144-021-02689-1 PMC8743689 35035192

[bibr20-10664807221123550] Kaddatz J. (2020, April 9). Families struggle to cope with financial impacts of the COVID-19 pandemic. *The Vanier Institute of the Family*. https://vanierinstitute.ca/families-struggle-to-cope-with-financial-impacts-of-the-covid-19-pandemic/

[bibr23-10664807221123550] Laufer A. Bitton M. S. (2021). Parents' perceptions of children's behavioral difficulties and the parent-child interaction during the COVID-19 lockdown. *Journal of Family Issues, 0*(0), 1-20. 10.1177/0192513X211054460 PMC9922668 36818819

[bibr25-10664807221123550] Lee S. J. Ward K. P. (2020). Research brief: Stress and parenting during the coronavirus pandemic. *University of Michigan Parenting in Context Research Lab*. https://www.parentingincontext.org/stress-and-parenting-during-a-pandemic.html

[bibr26-10664807221123550] Lim T. S. H. Tan M. Y. Aishworiya R. Kang Y. Q. Koh M. Y. Shen L. Chong S. C. (2021). Factors contributing to psychological ill effects and resilience of caregivers of children with developmental disabilities during a nationwide lockdown during the COVID-19 pandemic. Journal of *Autism and Developmental Disorders, 52*(2022), 3015-3025. 10.1007/s10803-021-05201-7 PMC8264471 34236591

[bibr27-10664807221123550] Marzilli E. Cerniglia L. Tambelli R. Trombini E. De Pascalis L. Babore A. Trumello C. Cimino S. (2021). The COVID-19 pandemic and its impact on Families’ mental health: The role played by parenting stress, parents’ past trauma, and resilience. International Journal of Environmental Research and Public Health, 18(21), 11450. 10.3390/ijerph182111450 34769967 PMC8583183

[bibr28-10664807221123550] Munoz R. T. Brady S. Brown V. (2017). The psychology of resilience: A model of the relationship of locus of control to hope among survivors of intimate partner violence. Traumatology, 23(1), 102–111. 10.1037/trm0000102

[bibr29-10664807221123550] Ontario Ministry of Education (2021, April 21). *Education Facts*, *2019-2020.* http://www.edu.gov.on.ca/eng/educationfacts.html

[bibr31-10664807221123550] Patton M. Q. (2005). Qualitative Research. Sage.

[bibr32-10664807221123550] Prime H. Wade M. May S. S. Jenkins J. M. Browne D. T. (2021). The COVID-19 family stressor scale: Validation and measurement invariance in female and male caregivers. *Frontiers in Psychiatry, 12*(1), 1-11. 10.3389/fpsyt.2021.669106 PMC8193227 34122184

[bibr33-10664807221123550] Prime H. Wade M. Browne D. T. (2020). Risk and resilience in family well-being during the COVID-19 pandemic. American Psychologist, 75(5), 631–643. 10.1037/amp0000660 32437181

[bibr34-10664807221123550] Qualtrics (2022). Qualtrics, Provo, Utah, USA. Available at: https://www.qualtrics.com

[bibr37-10664807221123550] Rodrigues M. Silva R. Franco M. (2021). COVID-19: Financial stress and well-being in families. Journal of Family Issues, 1–22. 10.1177/0192513X211057009 PMC10090962 37064997

[bibr38-10664807221123550] Romero E. López-Romero L. Domínguez-Álvarez B. Villar P. Gómez-Fraguela J. A. (2020). Testing the effects of COVID-19 confinement in Spanish children: The role of Parents’ distress, emotional problems and specific parenting. International Journal of Environmental Research and Public Health, 17(19), 6975. 10.3390/ijerph17196975 32987641 PMC7578923

[bibr39-10664807221123550] RStudio Team (2020). RStudio: Integrated Development for R. RStudio, PBC. http://www.rstudio.com/

[bibr40-10664807221123550] Sorkkila M. Aunola K. (2021). Resilience and parental burnout among Finnish parents during the COVID-19 pandemic: Variable and person-oriented approaches. *The Family Journal, 30*(2), 139–147. 10.1177/10664807211027307 PMC8980848 35399756

[bibr41-10664807221123550] Statistics Canada (2020, July 9). Impacts of COVID-19 on Canadian families and children. *Government of Canada*. https://www150.statcan.gc.ca/n1/daily-quotidien/200709/dq200709a-eng.htm

[bibr42-10664807221123550] Statistics Canada (2021, March 23). Canadian Income Survey, 2019. *Government of Canada*. https://www150.statcan.gc.ca/n1/daily-quotidien/210323/dq210323a-eng.htm

[bibr43-10664807221123550] Subramanian S. (2021, June 4). The lost year in education. *Maclean*’*s*. https://www.macleans.ca/longforms/covid-19-pandemic-disrupted-schooling-impact/

[bibr44-10664807221123550] Szpunar M. Vanderloo L. M. Bruijns B. A. Truelove S. Burke S. M. Gilliland J. Irwin J. D. Tucker P. (2021). Children and parents' perspectives of the impact of the COVID-19 pandemic on Ontario children's physical activity, play, and sport behaviours. *BMC Public Health, 21*(2021), 2271, 1-17. 10.1186/s12889-021-12344-w PMC8666344 34903197

[bibr47-10664807221123550] Wang X. Cheng Z. (2020). Cross-sectional studies: Strengths, weaknesses, and recommendations. Chest, 158(1), 65–71. 10.1016/j.chest.2020.03.012 32658654

[bibr48-10664807221123550] Weaver J. L. Swank J. M. (2020). Parents’ lived experiences with the COVID-19 pandemic. The Family Journal, 29(2), 136–142. 10.1177/1066480720969194

[bibr49-10664807221123550] World Health Organization (2020, April 27). *WHO Timeline – COVID-19*. https://www.who.int/news/item/27-04-2020-who-timeline---covid-19

[bibr50-10664807221123550] Ye Z. J. Qiu H. Z. Li P. F. Chen P. Liang M. Z. Liu M. L. Yu Y. L. Wang S. N. Quan X. M. (2017). Validation and application of the Chinese version of the 10-item Connor-Davidson resilience scale (CD-RISC-10) among parents of children with cancer diagnosis. European Journal of Oncology Nursing: The Official Journal of European Oncology Nursing Society, 27(1), 36–44. 10.1016/j.ejon.2017.01.004 28279394

